# Rigid motion-resolved B_1_^+^ prediction using deep learning for real-time parallel-transmission pulse design

**DOI:** 10.1002/mrm.29132

**Published:** 2021-12-27

**Authors:** Alix Plumley, Luke Watkins, Matthias Treder, Patrick Liebig, Kevin Murphy, Emre Kopanoglu

**Affiliations:** 1 School of Psychology, Cardiff University Brain Research Imaging Centre (CUBRIC), Cardiff University, Cardiff, United Kingdom; 2 School of Physics & Astronomy, Cardiff University Brain Research Imaging Centre (CUBRIC), Cardiff, United Kingdom; 3 School of Computer Science and Informatics, Cardiff University, Cardiff, United Kingdom; 4 Siemens Healthcare, GmbH, Erlangen, Germany

## Abstract

**Purpose:**

Tailored parallel-transmit (pTx) pulses produce uniform excitation profiles at 7 T, but are sensitive to head motion. A potential solution is real-time pulse redesign. A deep learning framework is proposed to estimate pTx B_1_^+^ distributions following within-slice motion, which can then be used for tailored pTx pulse redesign.

**Methods:**

Using simulated data, conditional generative adversarial networks were trained to predict B_1_^+^ distributions in the head following a displacement. Predictions were made for two virtual body models that were not included in training. Predicted maps were compared with groundtruth (simulated, following motion) B_1_^+^ maps. Tailored pTx pulses were designed using B_1_^+^ maps at the original position (simulated, no motion) and evaluated using simulated B_1_^+^ maps at displaced position (ground-truth maps) to quantify motion-related excitation error. A second pulse was designed using predicted maps (also evaluated on ground-truth maps) to investigate improvement offered by the proposed method.

**Results:**

Predicted B_1_^+^ maps corresponded well with ground-truth maps. Error in predicted maps was lower than motion-related error in 99% and 67% of magnitude and phase evaluations, respectively. Worst-case flip-angle normalized RMS error due to motion (76% of target flip angle) was reduced by 59% when pulses were redesigned using predicted maps.

**Conclusion:**

We propose a framework for predicting B_1_^+^ maps online with deep neural networks. Predicted maps can then be used for real-time tailored pulse redesign, helping to overcome head motion–related error in pTx.

## 1 Introduction

Parallel transmission (pTx) of RF pulses through independently controlled channels can help to overcome B_1_ nonuniformity seen in the head at 7 T,^1, 2^ particularly when tailored pulses are used.^3^ Tailored pulse design incorporates the measured transmit sensitivities (B_1_^+^) of each pTx channel, achieving a homogeneous flip angle across specified slices or regions. For optimal tailored pulse performance, the measured B_1_^+^ distributions must match those present at the time of pulse playout. However, channels’ electromagnetic fields (including B_1_^+^) and their interference patterns depend critically upon the object being imaged (i.e., the coil load), including its position, geometry, and composition.^4-6^

Geometrical and compositional differences between human subjects are partly addressed in alternative, nontailored approaches such as universal pulses (UPs),^7, 8^ SmartPulse,^9^ and fast online-customized pTx pulses.^10^ Intersubject robustness is achieved by designing a UP (offline) to minimize error across a small database of representative subjects. An underlying assumption is that the range in head geometry and composition across human subjects is relatively constrained, implying that B_1_^+^ distributions are similarly constrained. The designed pulse (a minimum error solution for excitation over multiple B_1_^+^ distributions) is therefore assumed to work fairly well for any individual subject without the need for B_1_^+^ mapping. Plug-and-play usability of UPs in pTx has led to the method’s growing popularity.

However, the intersubject robustness of UPs comes at a cost to flip-angle uniformity. Tailored pulses typically yield lower normalized RMS error (nRMSE) of flip angle compared with UPs (7% vs 11% in Gras et al^7^). Additionally, the database approach is problematic in cases in which an individual is an outlier with respect to anatomies represented in the database. Moreover, these methods do not address the dependence of B_1_^+^ on load position, leading to unpredictable pulse performance in cases of different initial subject positioning^11^ and/or within-scan head motion.^12-14^ The former is often overlooked, whereas the latter is commonly reported.^15^ Large head movements (exceeding 20 mm/degree) often occur among certain clinical populations,^16, 17^ elderly,^18^ and pediatric^19, 20^ subjects. Because flip angle (and therefore the acquired signal) depends on B_1_^+^, displacements of approximately 5° have been found to cause an excitation error of 12%–19% (percent of target flip angle) when using pTx at 7 T,^12^ with larger movements causing larger flip angle–related artifacts.

A few approaches have been proposed to correct motion-related RF field changes. Faraji-Dana et al. partially overcame motion-related effects on the (receive) B_1_ field by simply reorienting coils’ measured sensitivity maps using a Euclidean transformation.^21^ Similarly, Wallace et al. used radial basis functions to extrapolate channel sensitivities to voxel locations outside of the head, providing sensitivity information for all voxels in the FOV, regardless of head position.^22^ Extrapolated maps were used for retrospective correction. Neither approach considered dynamic motion-related field changes (e.g., changes in coil loading, shifting susceptibility gradients in tissue), as their effects were deemed minimal at 3 T. However, interactions between channels’ highly nonuniform transmit fields at 7 T,^23^ especially with pTx, indicate that dynamic motion-induced field changes cannot be overlooked. In contrast with these approaches, data-driven approaches inherently incorporate these changes.

Motion artifacts are often addressed through retrospective correction^22, 24-27^; however, this is problematic for several reasons. First, the issues described previously cannot be corrected retrospectively without motion-resolved B_1_^+^ maps, which are not available. Second, channels’ electric fields depend on the same factors, including load position. Specific absorption rate (SAR) distribution and associated tissue heating are therefore also sensitive to motion, and are especially so in pTx due to constructive interference between channels’ electric fields.^28-30^ Peak local SAR can exceed safety limits when head motion occurs in pTx simulations^29^—a critical issue that cannot be addressed retrospectively. Conservatively bounded SAR estimates may be used, but this can prevent optimal imaging performance by limiting the RF power.^2, 11, 31^ In this study, the effect of motion on flip angle is the primary focus.

It is therefore desirable to overcome the motion dependence of tailored pTx pulse performance, and to do so using prospective techniques. Real-time pTx pulse design has been proposed as a solution, in which channels’ complex coefficients are continuously updated to counteract motion-induced sensitivity changes. Multispoke pTx pulses can be designed in less than 0.5 seconds,^32^ whereas 2D spatially selective spiral pulses can be estimated in about 9 ms using deep neural networks.^33^ With motion detection (e.g., Refs 22, 25, 34, and 35), channel updates could be determined by instantaneous head position, retaining flip-angle uniformity in cases of arbitrary and/or extreme motion. However, the required updates to channel coefficients depend on the motion-related field changes. Because real-time (i.e., motion-resolved) B_1_^+^ maps are not measurable, this requires that the relationship between head position and B_1_^+^ distribution to be characterized.

Deep convolutional neural networks have previously been used to estimate (non-pTx) B1^+^ distributions. In Wu *et al*., ^36^ high-quality maps were predicted from reconstructed T_1_-weighted images, removing the need for B_1_^+^ mapping, while still allowing retrospective correction of B_1_^+^ related artifacts in quantitative MRI. This approach was limited to postprocessing; prediction quality deteriorated when B_1_^+^ was predicted directly from undersampled images. Abbasi-Rad et al. used a convolutional neural network to reconstruct B_1_^+^ from a localizer scan for the purpose of SAR reduction through pulse scaling based on slice-wise B_1_^+^ magnitude; however, B_1_^+^ prediction quality was dependent on head position.^37^

In this work, we train a system of conditional generative adversarial networks^38^ to predict pTx B_1_^+^ distributions (referred to as B_1_ maps) following simulated head motion, given the initial B_1_ maps at the centered position as input. If used in conjunction with motion detection, this would constitute motion-resolved B_1_-map estimation, and therefore permit real-time tailored pulse design. B_1_-map prediction quality is assessed by comparison with ground-truth (simulation output) B1 maps following motion. Furthermore, flip-angle distributions of multispoke pTx pulses designed using network-predicted B1 maps are compared with those produced by tailored pulses designed using the initial subject-specific B1 maps alone. Finally, we also observe peak 10-g averaged local SAR for both pulses following motion.

## 2 Methods

### 2.1 Simulations and data

**Figure 1:**
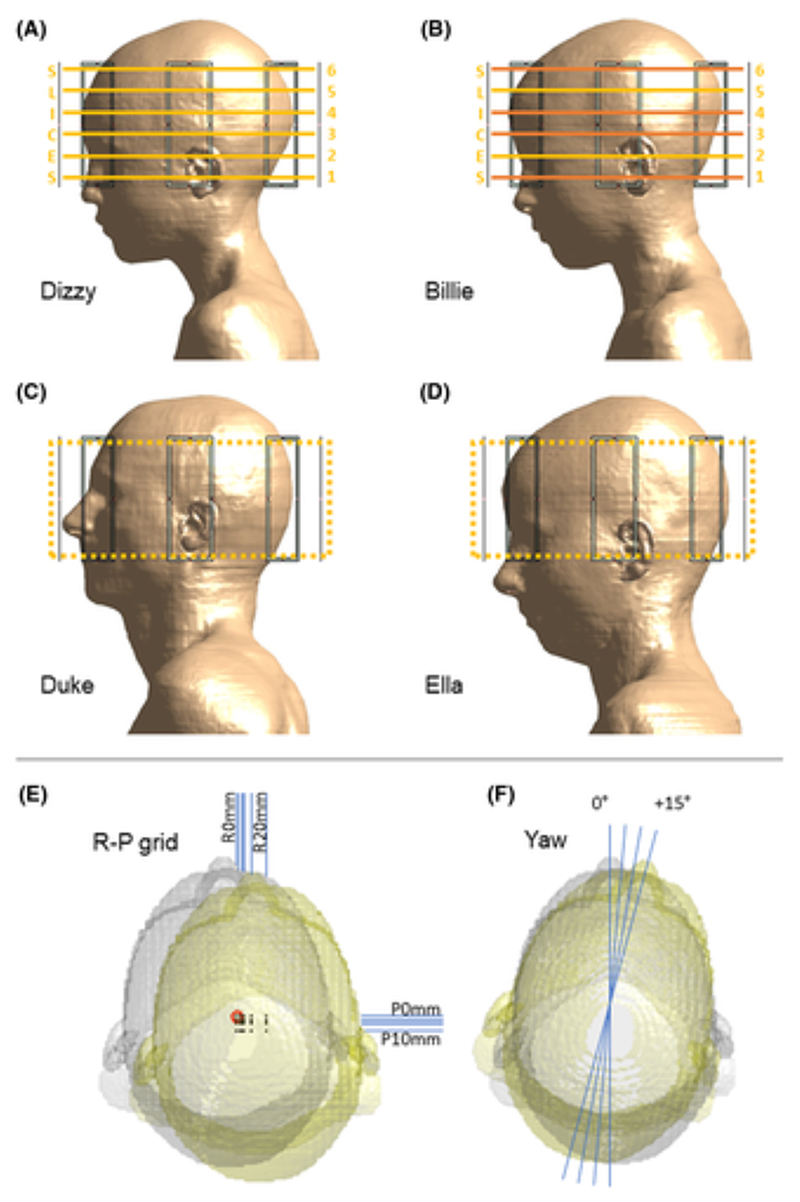
Simulation model setup. (A–D) The four body models used in Sim4Life simulations. Ella and Duke (C, D) were used to generate training data for networks, Billie (B) was used for network validation and testing, and Dizzy (A) was used for testing only. Testing (including pulse design) was conducted at the six indicated slice locations. Validation slices were offset by about 4 mm from these, but within the same axial range. Slices in orange were also used for specific absorption rate (SAR) evaluations. All axial slices within the dashed slab were used for training. (E) Positions simulated for the R-P grid. The origin of the central position is indicated with a red circle, whereas all other positions’ origins are indicated with black dots. Axial displacements were all possible combinations of rightward (R) 0, 2, 4, 5, 10, and 20 mm and posterior (P) 0, 2, 4, 5, and 10 mm. (F) Yaw rotations were 5°, 10°, and 15°. The head at the central position (gray isosurface) and most extreme displaced position (yellow isosurface) are shown.

Dizzy, Billie, Duke, and Ella (Figure 1A-D) of the Virtual Population^39^ (IT’IS, Zurich, Switzerland) were simulated with a generic 8-channel pTx coil in Sim4Life (ZMT, Zurich, Switzerland). Each model was simulated at one central, and 32 off-center, positions. Off-center positions included rightward 2, 4, 5, 10, and 20 mm, posterior 2, 4, 5, and 10 mm, and all possible combinations thereof. These 29 positions are hereafter referred to as the R-P grid (Figure 1E). In addition, yaw 5°, 10°, and 15° positions were also simulated (Figure 1F). The Duke model was scaled to 90% of the original size, as the body and coil models intersected at some positions when the model was full-sized. To ensure consistent voxelization (and therefore consistent partial volume effects) in the body model across all simulated positions, the coil array was displaced rather than the body model. Simulations included the head, neck and shoulders,^40^ and were run at 295 MHz following coil tuning to this frequency. Simulation results were normalized to an accepted power of 1 W per channel beyond the input port to the coil elements, to override imperfections in coil matching and any positional dependencies. The simulations were manually checked for input impedance and reflection coefficient as well as field smoothness across positions.

Channels’ 3D B_1_, electric field, current density, and SAR distributions were masked to exclude background (air) voxels and exported to MATLAB (The MathWorks, Natick, MA). To incorporate interactions between channels for local SAR evaluations, 10*g*-averaged Q-matrices were calculated.^28, 41, 42^ Elements of the 8 × 8 Q-matrices were

(1) $$Q_{ij}(r)=\frac{1}{2\rho (r)}\left[J_{x,j}^{H}(r)E_{x,i}(r)+J_{y,j}^{H}(r)E_{y,i}(r)+J_{z,j}^{H}(r)E_{z,i}(r)\right],$$

where *ρ*(*r*)is the tissue mass density (kg/m^3^) in voxel *r*; *j* is the complex current density (A/m^2^); *E* is the complex electric field (V/m); *x*,*y* and are the three Cartesian axes; *i*and *j* are transmit channel indices; and *H* denotes Hermitian transpose.

B_1_ maps from 51 slices spanning a mid-axial slab with a thickness of 9 cm from the Duke and Ella body models (Figure 1C,D) were prepared for network training by interpolating to 256 × 256 in-plane resolution. The same preprocessing was applied to the Billie and Dizzy data, but at only six slice locations (Figure 1A,B). Magnitude and phase data were separated and normalized between 0 and 1, where 1 corresponds to the maximum magnitude across all channels, slices, and body models, and to 2π for phase. Random offsets were applied to phase maps so that the phase wrap boundary did not occur at the same location across slices. B_1_ maps were input to networks as individual axial slices with size 256 × 256 × 8, where the third dimension is channels. Corresponding B_1_-map slices before (input) and after (ground truth) a given displacement formed the networks’ input-target pairs. Note that inputs are not necessarily at the centered position (explained later in Section 2.2).

### 2.2 Neural networks and network training

**Figure 2:**
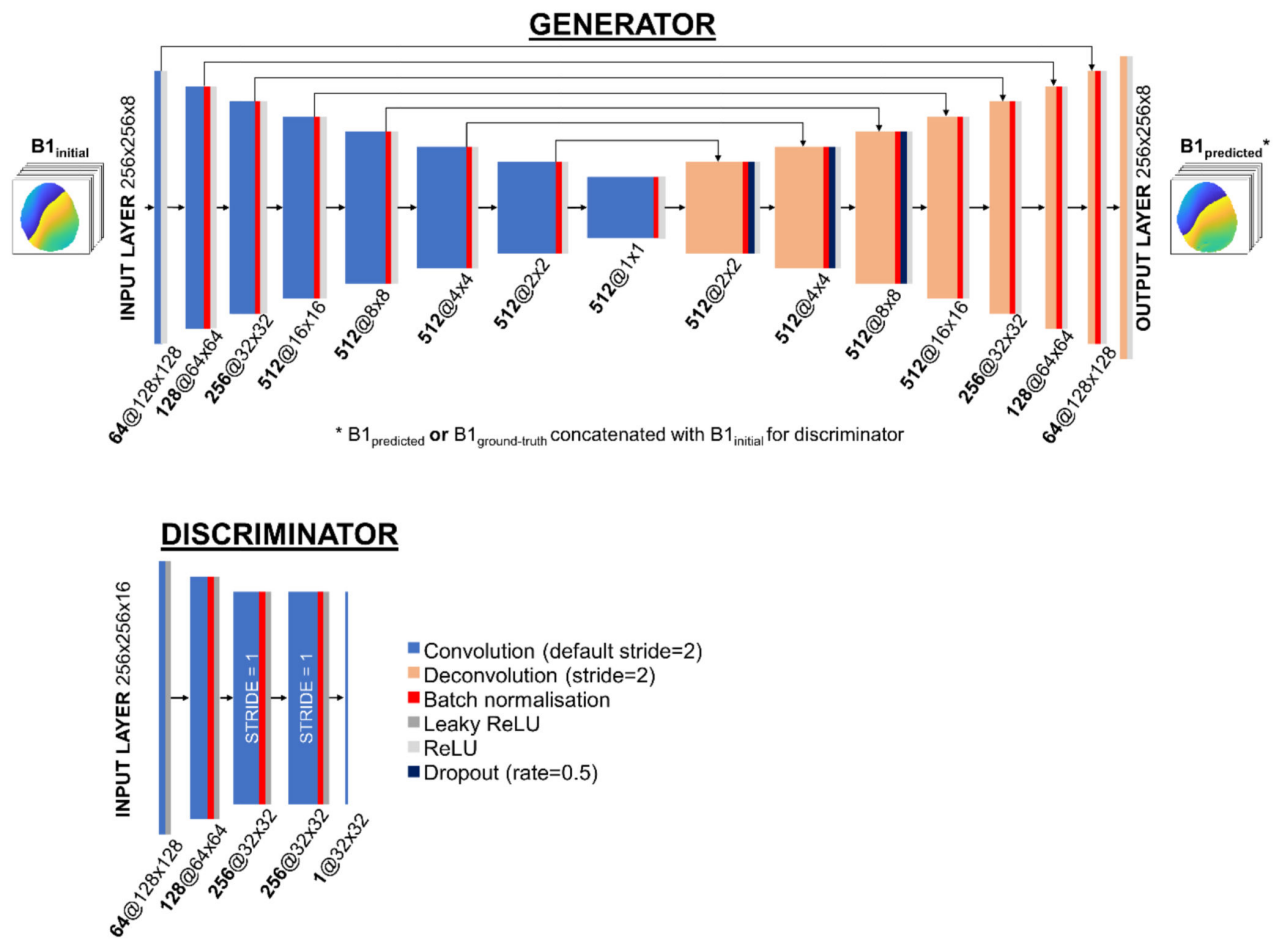
Conditional generative adversarial network (cGAN) architecture. Generators were U-Nets with eight convolution and eight deconvolution layers, each with rectified linear unit (ReLU) activation. Discriminators consisted of five convolutional layers with ReLU activation. Square matrix size and number of filters (initially 64 for phase networks) are indicated beneath the layers. Convolution stride was 2 except where specified. Skip connections are shown with arrows. Dropout was applied at indicated layers (dark blue). Batch normalization (red) was used for phase networks, but not for magnitude networks. Filters for phase networks were 8 × 8. Magnitude networks used double the number of filters, with filter size = 4 × 4.

Models were implemented in TensorFlow 2.3^43^ using Python 3.7. Network architecture is summarized in Figure 2. Except where specified, network hyperparameters were the same as those used in the Pix2Pix conditional generative adversarial network.^44^ The generators were U-Net^45^ models with eight convolutional (encoding) and eight deconvolutional (decoding) layers linking the input and output (predicted) B_1_ maps, each followed by rectified linear unit activation layers. Filters were 4 × 4 for magnitude and 8 × 8 for phase. Although comprehensive hyperparameter optimization was beyond the scope of this project, during initial testing it was found that phase networks benefited from the large receptive field of 8 × 8 filters. Conversely, magnitude networks generated smoother maps when more filters were used. To avoid increasing the number of trainable parameters, filters were smaller for magnitude. The number of filters (initially 128 for magnitude, and 64 for phase) increased to a maximum of 1024 (512 for phase) for the middle layers, and stride size was 2. Filters were split into eight groups to facilitate simultaneous processing of all pTx channels. For phase, batch normalization was applied at all layers except the first convolution layer. For magnitude, removing batch normalization resulted in a smoother training curve and higher-quality estimated maps. Skip connections joined each convolution layer to the symmetric deconvolution layer for network stability. The network was regularized through dropout layers following each of the first three deconvolution layers (rate = 0.5).

In contrast to encoder–decoder models that typically rely on minimizing L1 loss between predicted and target images, generative adversarial networks include an additional loss term, which helps to reduce blurring often seen with L1 loss alone.^44^ This is provided through a second convolutional neural network—the discriminator—which is trained to distinguish between generator-predicted and ground-truth distributions. The input B1 maps, concatenated with either ground-truth or generator-predicted B1 maps, served as input to the discriminators, which consisted of five convolution layers. The discriminators used leaky rectified linear unit activation layers (*a* = 0.3) as recommended in Radford et al.^46^ Filter size was the same as that for the generators, and convolution stride was 2 except for the final two layers, where it was 1. A single 2D distribution of probability (entropy) values was output.

The overall conditional generative adversarial network loss function can be expressed as

(2) $$\;Loss\;=\arg\underset{G}{\min}\;\underset{D}{\max}L_{cGAN}(G,D)+\lambda L_{L1}(G)$$

where *G* denotes the generator; *D*is the discriminator; and *λ* (set to 100) is a scaling parameter acting on the L1-norm between generator-predicted and ground-truth maps. The first term can be further described as

(3) $$\begin{array}{l} L_{cGAN}(G,D)=E_{B1_{gt},B1_{pedicted\;}}\left[\log D\left(B1_{gt},B1_{predicted\;}\right)\right]\\ +E_{B1_{gt},},B1_{initial}[\log\left(1-D\left(B1_{gt},G\left(B1_{gt},B1_{initial\;}\right)\right)\right],\; \end{array}$$

where B1_gt_ are the ground-truth displaced B_1_ maps; B1_predicted_ are the generator-predicted displaced B_1_-maps; and B1_initial_ are the pre-displacement B_1_ maps (network input).

The effect of head motion on B_1_ depends on the displacement type (i.e., direction, magnitude).^12, 13^ Because data-driven approaches assume that all input-target pairs share a common underlying mapping, separate networks were trained for different displacement types (e.g., rightward vs posterior). Head motion was discretized into large (5 mm) and small (2 mm) displacements in rightward (R) and posterior (P) directions to cover the R-P grid. Additional networks were trained for 5° yaw rotation. Separate networks were trained for magnitude and phase, yielding a total of 10 networks.

The Duke and Ella data were used for training. All available relative displacements were included. For example, to train the R5 mm network, such as (input)–(target) pairs included (R0, P0 mm)–(R5, P0 mm); (R5, P0 mm)–(R10, P0 mm); (R5, P2 mm)–(R10, P2 mm). This yielded a training data set of 1020 unique slices for rightward and leftward networks, and 1224 for each posterior network. The yaw network training database was smaller (306 slices).

The Adam^47^ optimizer was used to train models for 60 epochs. Learning rate was critical during initial testing, so learning rates within the range 5e-5 to 1e-3 were tested. The default value of 2e-4 converged most effectively and was therefore used for all networks. Network weights were saved at the epoch, which yielded the lowest total error across the validation data set (the Billie data) as a form of early stopping to help prevent network overfitting. Networks took approximately 16 hours to train with a batch size of 1 using a standard PC with NVIDIA GeForce GTX 1050 Ti.

### 2.3 Network evaluation and cascading

Networks were tested using the Billie and Dizzy data at six slice locations (Figure 1A). For Billie, different slices were used compared with those used for early stopping (Dizzy was not involved in the training process at all). Like training, testing was conducted for all available examples of each displacement, yielding test data sets of between 6 and 72 slices. In addition to the positions listed in section 2.1, Billie and Dizzy models were simulated at three combined yaw-rightward positions to test performance for motion involving both rotation and translation. Because networks were only trained for five displacements but evaluated at 35 positions, networks were cascaded where necessary. Starting with the center position’s B_1_ maps as input, generators were run sequentially, with the output of one generator used as input to the next, until the desired evaluation position was reached. For example, R5 mm, R5 mm, and P2 mm networks were cascaded for evaluation at the (R10, P2 mm) position.

Finally, the Billie model was also simulated at inferior 5, 10, and 15 mm to investigate error for through-plane motion.

Predicted B_1_ maps were exported to MATLAB. Voxels with < 1% of the maximum magnitude were smoothed with a Gaussian kernel. Corresponding magnitude and phase network outputs were subsequently combined to form complex predicted maps (B1_predicted_).

The B1_predicted_ quality was assessed through voxel-wise correlation (using MATLAB’s corrcoef function) and prediction error between predicted and ground-truth maps at each position. These values were compared with error and correlation following head motion (i.e., between the two simulated maps). Calculations were performed on the tissue-masked region, with the outermost two voxels excluded to avoid artificial amplification of error due to partially filled voxels. Prediction error for magnitude and phase distributions were assessed through nRMSE and L1 norm, respectively, as follows:

(4) $$nRMSE_{∥prediction\;∥}=100\times \frac{\sqrt{\frac{1}{N_{v}}\sum_{r=1}^{N_{v}}∥B1_{gt}(r)-B1_{predicted}(r){∥}^{2}}}{\frac{1}{N_{v}}\sum_{r=1}^{N_{v}}∥B1_{gt}(r)∥}$$

(5) $$L1_{∠prediction\;}=\frac{1}{N_{v}}\sum_{r=1}^{N_{v}}∥∠e^{j\left(B1_{gf}(r)-B1_{predicted\;}(r)\right)}∥$$

where *j* is $\sqrt{-1}$; and *N_υ_* is the number of voxels in a slice, indexed by *r*. Motion-induced error was calculated analogously, but substituting B1_initial_ for B1_predicted_ in Equations 4 and 5.

### 2.4 Pulse design and analysis

**Figure 3:**
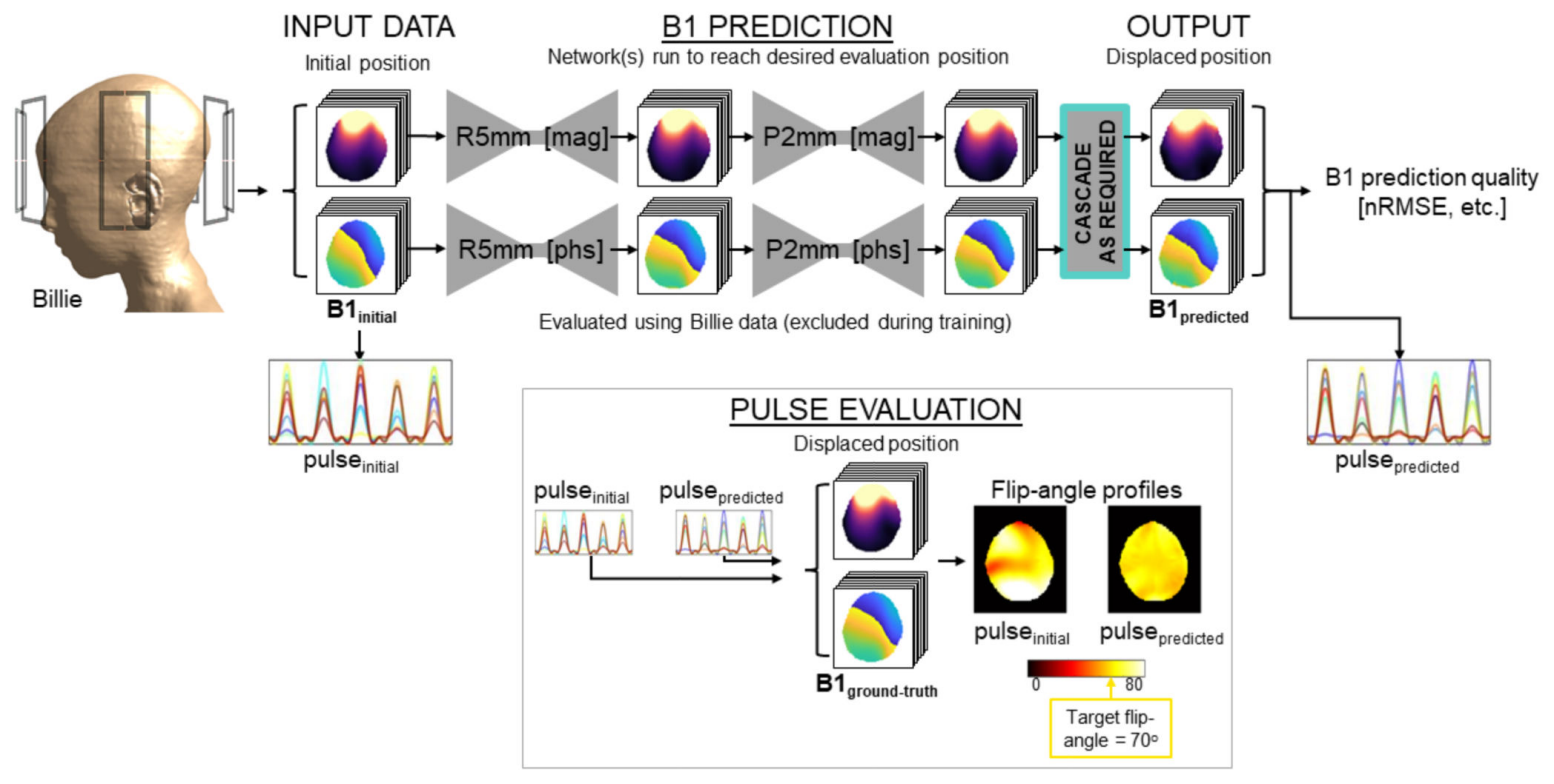
Outline of the testing workflow. Simulated B_1_^+^ maps from the center position are input to the first trained generator. Generators were trained for small displacements but can be run sequentially (cascaded) until the desired off-central (displaced) position is reached in evaluations. Prediction quality is assessed by normalized RMS error (nRMSE) and voxelwise correlation with respect to the ground-truth (simulation output) displaced B_1_^+^ map. In addition, pulses designed using the initial B_1_^+^ map are compared with those designed using predicted maps, in terms of their excitation profiles following head motion.

Outputs from the R-P grid positions were further processed to assess whether predicted maps were of sufficient quality to be used for tailored pTx pulse design. Five-spoke excitation pulses were designed using a small tip-angle spatial domain method,^3, 48, 49^ and two approaches were compared in terms of their performance following motion within the R-P grid. A schematic of the process is shown in Figure 3. First, a conventional tailored pulse (pulseinitial) was designed using the subject-specific B_1_ maps at the initial position (B1_initial_). A uniform magnitude target excitation profile (target flip angle = 70°) was specified for pulse_initial_. Pulse coefficients were optimized iteratively to minimize magnitude error, whereas the profile’s phase was relaxed.^50^ The resultant complex profile was used as the target profile for a second pulse (pulsere-designed), which was designed without phase relaxation (because magnitude and phase distributions need to be consistent across positions to ensure data consistency for motion occurring mid-acquisition). Pulser_e-designed_ was designed using the network-output B1_predicted_ (the proposed approach).

Both pulses (pulse_initial_ and pulsere-designed) were subsequently evaluated using the ground-truth B_1_ maps at the displaced position (B1_gt_) to quantify motion-induced effects on the conventional approach, and improvement provided by the proposed method. Their flip-angle distributions were compared with that of pulse_initial_ without motion in terms of nRMSE, expressed as percent target flip angle as follows:

(6) $$nRMSE_{\theta }=100\times \frac{\sqrt{\frac{1}{N_{v}}\sum_{r=1}^{N_{v}}∥\theta_{displaced\;}(r)-\theta_{initial\;}(r){∥}^{2}}}{\theta_{t}}$$

where *θ_initial_* is flip angle without motion; *θ_displaced_* is that following motion (with either pulse_initial_ or pulse_re-designed_); and *θ_t_* is the target flip angle. The nRMSE for pulse_initial_ without motion (i.e., the “gold standard”) was also calculated by substituting *θ_displaced_* for *θ_t_* in Equation 6.

Peak local SAR (psSAR) of both pulses was also evaluated using the 10-g averaged Q-matrices at each position. Because psSAR sensitivity to motion has been reported to exhibit no slice dependence,^29^ SAR was evaluated at four target imaging slices (out of the six used for pulse design) (Figure 1A).

## 3 Results

### 3.1 B_1_ prediction quality

**Figure 4:**
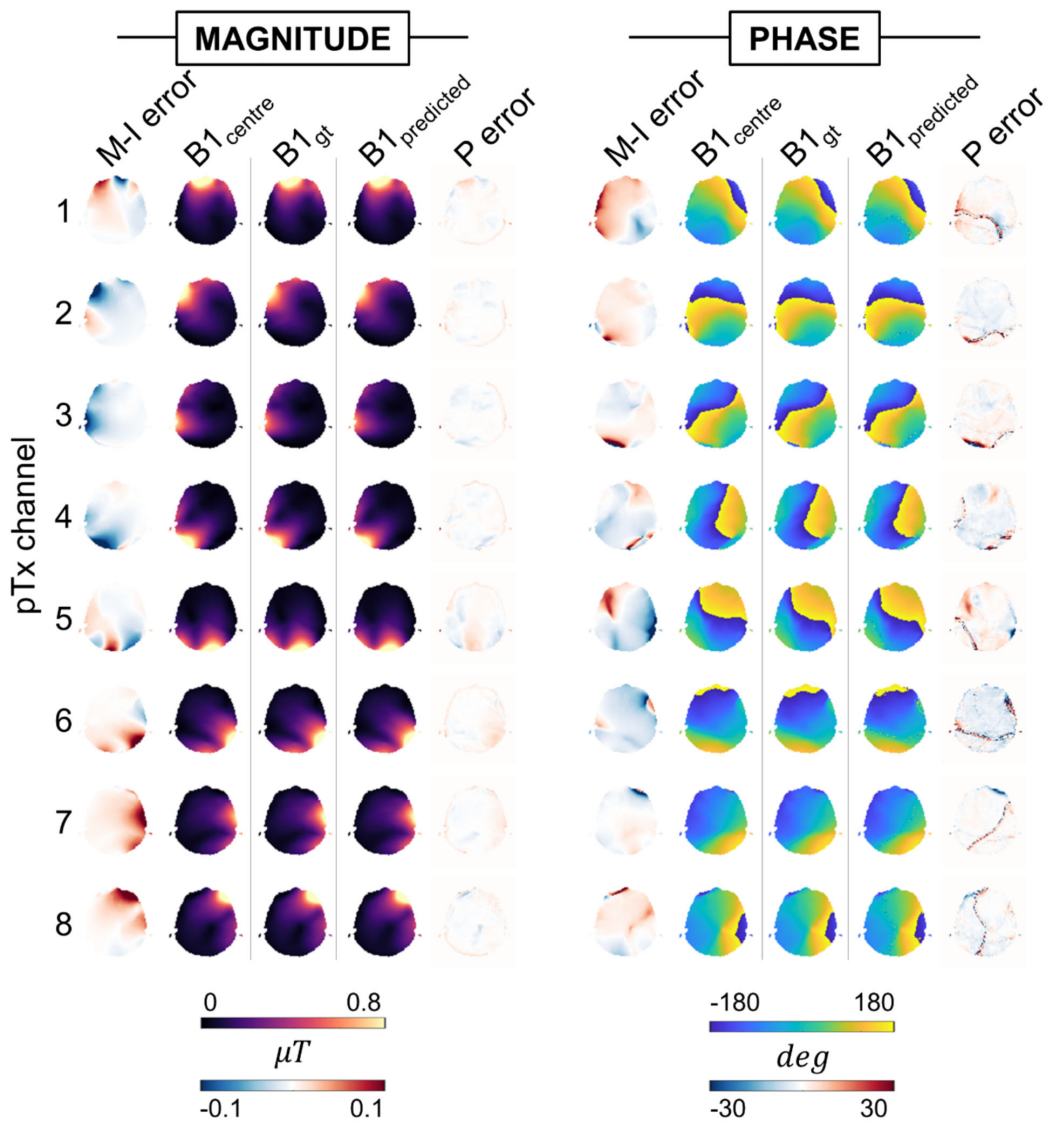
Example magnitude and phase B_1_^+^ maps and error following a rightward displacement of 5 mm (slice location = 2). Motion-induced (M-I) error shows difference between simulation-output B_1_ at the centered and displaced positions (B1_initial_ and B1_gt_, respectively). Prediction (P) error shows the difference between simulation-output B1_gt_ and generator-predicted B_1_ (B1_predicted_). Motion-induced error (averaged across channels) for this example was 15.1% (magnitude) and 4.9° (phase), whereas mean prediction error was 3.2% (magnitude) and 3.5° (phase). Abbreviation: pTx, parallel transmission.

B_1_ maps were predicted by networks in about 14 ms using the same PC as used for training. Example B_1_ maps, motion-induced error, and prediction error are shown for a 5 mm displacement in Figure 4. Motion-induced error (averaged across channels) for this example was 15.1% (magnitude) and 4.9° (phase), whereas mean prediction error was 3.2% (magnitude) and 3.5° (phase).

**Figure 5:**
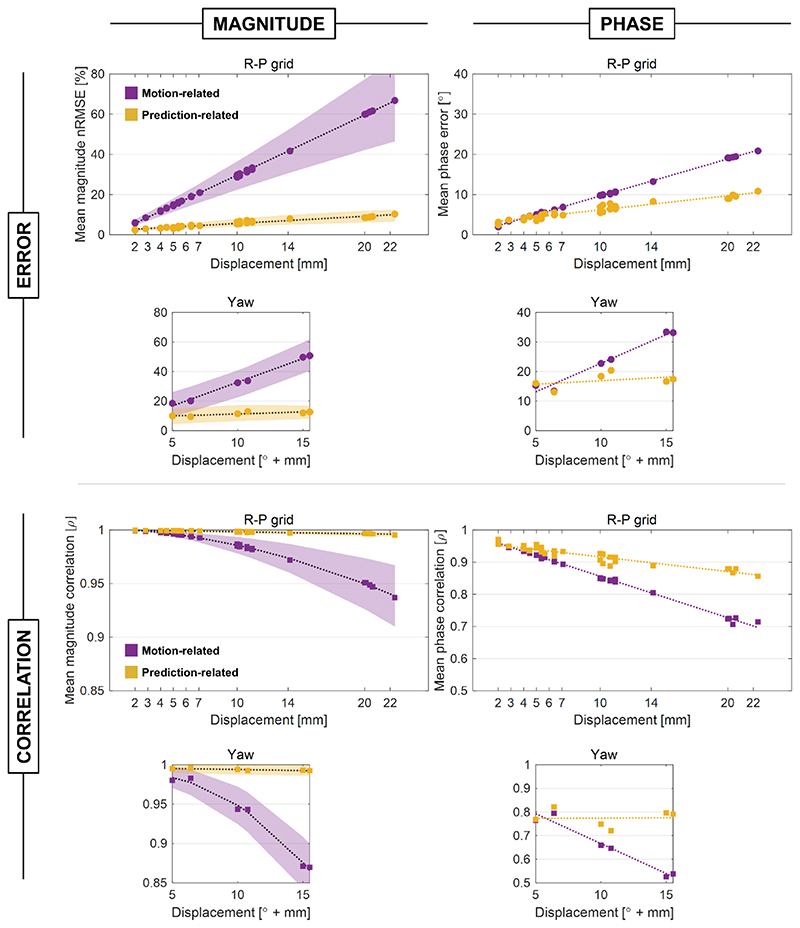
Error (nRMSE for magnitude, L1 norm for phase) and correlation coefficient (ρ) shown for magnitude and phase, averaged over Dizzy and Billie body models, channels, slices, and initial positions for each evaluated displacement. Translational displacements (the R-P grid) are shown in the large panels, while rotations (yaw) and combined rotation-translations (yaw plus a 4-mm translation) are shown in the smaller panels below (for the purpose of the x-axis, the amount of yaw rotation is treated as magnitude displacement; for example, yaw 5° plus 4-mm translation is shown at x = 6). The effects of motion are shown in purple, while network-related prediction error is shown in yellow. The SD is shown as shaded regions for magnitude but is omitted for phase for clarity, as values were similar.

Figure 5 shows a summary of error and correlation coefficient for magnitude and phase at each evaluated displacement (averaged across Dizzy and Billie models, slices, channels, and initial positions). Position dependence of prediction quality was minimal compared with motion-related error, as seen by the reduced gradient with respect to displacement norm in all cases. Dizzy and Billie models yielded very similar prediction quality (Supporting Information Figure S1).

**Figure 6:**
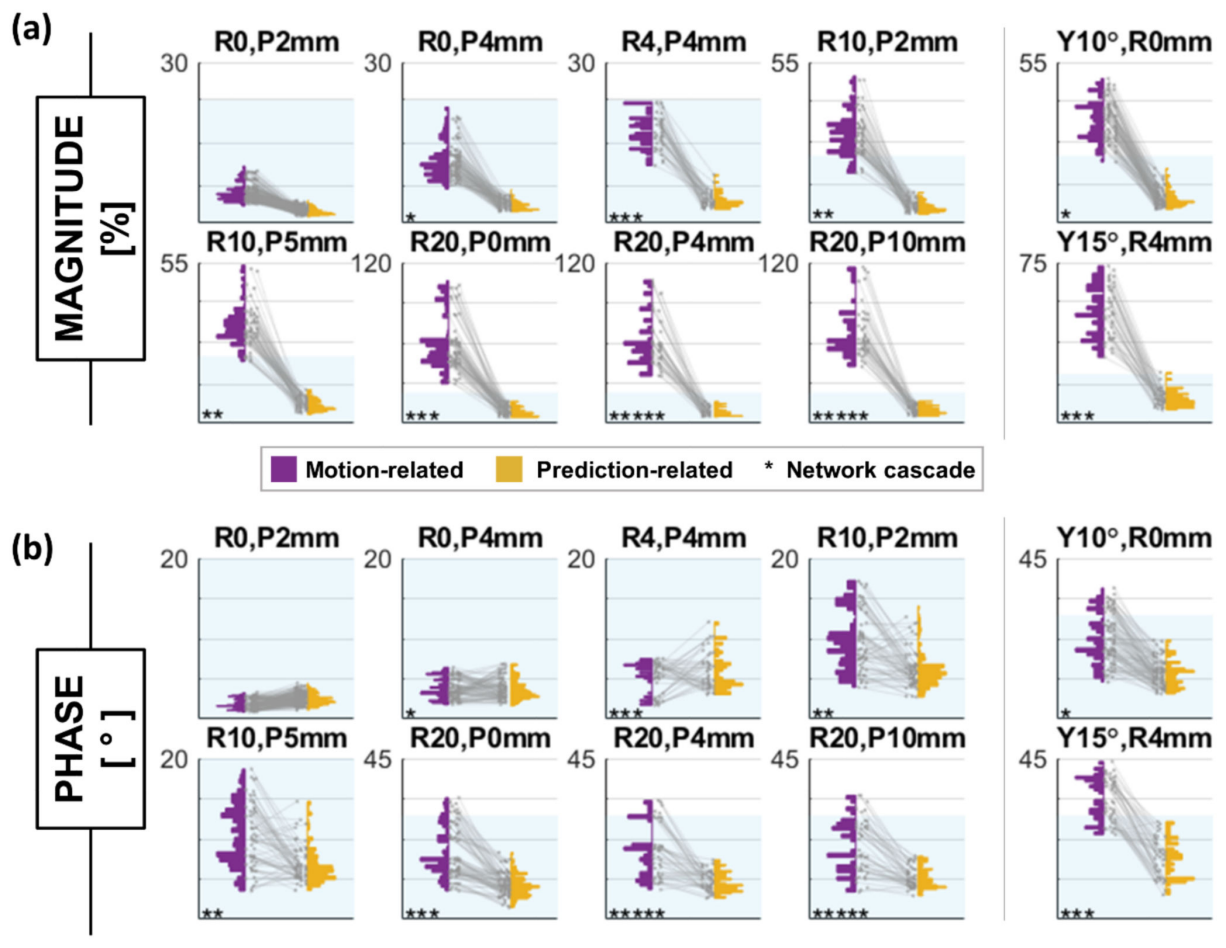
B_1_ error (nRMSE for magnitude [A], L1 norm for phase [B]) for all evaluations with the Billie model following 10 example displacements. Motion-related error is shown in purple, while error for predicted maps is in yellow. Asterisks indicate the number of network cascades required for evaluation. The blue-shaded region shows the maximum observed prediction error across all 35 displacements for the Billie model (consistent across panels).

Mean motion-induced magnitude error scaled linearly with displacement magnitude at about 3% per millimeter (or 3.2% per degree of rotation), compared with 0.36% per millimeter (0.27% per degree) for prediction error. Prediction error was lower than motion-related error in 99.8% of translation, and 90% of rotation evaluations. Figure 6A shows B_1_ magnitude nRMSE for magnitude for all slices and channels for 10 example displacements. Motion caused a worst-case magnitude error of 117% following a displacement of R20, P10 mm, whereas maximum prediction error was 33% (at the y15°, R4 mm position). Worst-case prediction error from the R-P grid was lower (20% at the R20, P10 mm position).

**Figure 7:**
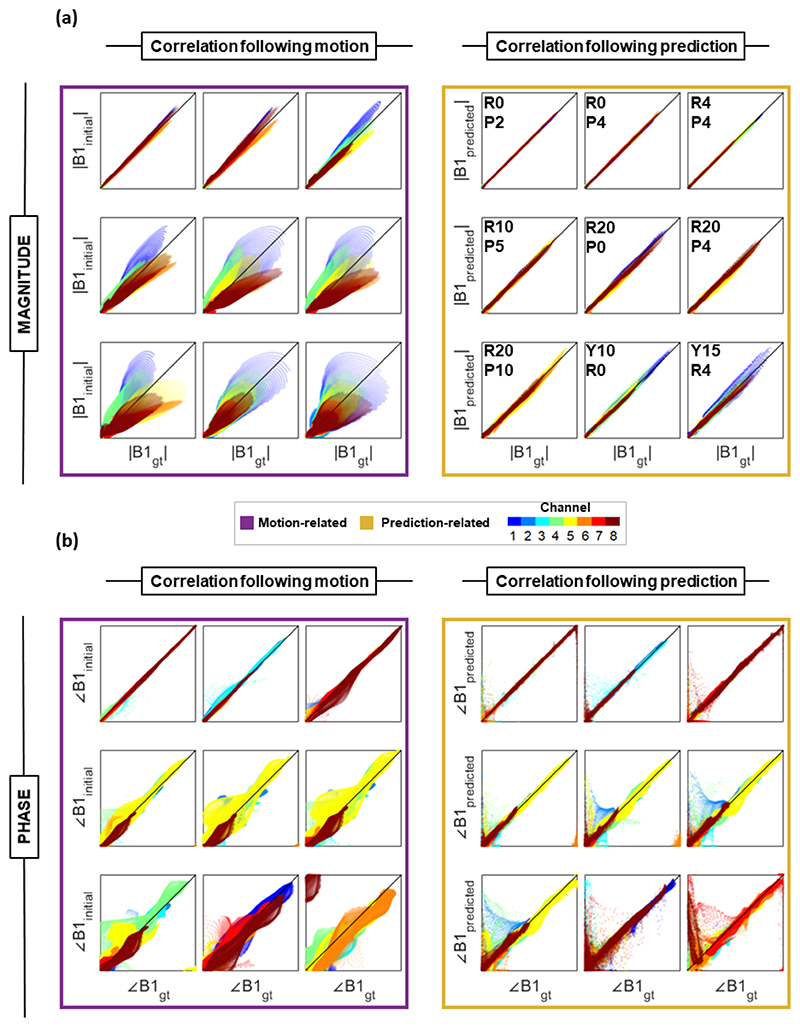
Example voxel-wise correlations between B1_initial_ and B1_gt_ (left) and B1_predicted_ and B1_gt_ (right) for nine example displacements. The pTx channels are indicated by color. The x and y axes range between 0 and 3 μT for magnitude (A), and 0 and 2π for phase (B).

Example magnitude correlations are shown in Figure 7A. The lowest observed correlation coefficient between B1_initial_ and B1gt magnitudes was 0.79 following a y15°, R4 mm displacement. Correlation between B1_predicted_ and B1gt did not fall below 0.96.

Motion-induced error and correlation were observed to be slice-dependent and channel-dependent (i.e., the error depended on the displacement relative to each channel’s location, as expected). The B1_predicted_ quality was similar across channels, as demonstrated by the strong correlation across all channels in Figure 7A. However, prediction error was somewhat slice-dependent, with the most inferior slice locations yielding the highest prediction errors (slice information not shown).

Phase error for 10 example displacements are shown in Figure 6B. For phase, maximum observed prediction error (57°) was similar to maximum motion-related error (55°). These worst cases arose in the Dizzy model; for the Billie model, maximum prediction error (29.4°) was lower than that caused by motion (44.2°). Furthermore, prediction error was lower than motion-induced error for phase in 68% and 66% of translation and rotation evaluations, respectively (including both models).

Yaw rotation caused substantially higher error than axial translations; for the R-P grid, maximum prediction-related and motion-related errors were 19.8° and 34.7°, respectively. Mean phase prediction error was less position-dependent than motion-related error, with axial translations causing error of approximately 0.9° per millimeter displacement, compared with 0.4° per millimeter in predicted maps. For displacements including rotation, analogous gradients were 2° and 0.2° per degree of yaw, respectively.

Mean phase correlation coefficient between predicted and ground-truth maps was higher than (or very similar to) that between initial and ground-truth maps for all displacements. Phase correlation examples are shown in Figure 7B. Correlation coefficient between B1_predicted_ and B1_gt_ exceeded that between B1_predicted_ and B1_initial_ in 69% of cases.

### 3.2 Parallel-transmit pulse performance

Subsequent analyses were conducted using the Billie model with the R-P grid data only. Five-spokes pTx pulses designed using B1_initial_ (pulse_initial_) yielded uniform flip-angle profiles (mean nRMSE ~1%) without motion. However as expected, uniformity was lost following axial translation. Pulses were about 7.7 ms long.

**Figure 8:**
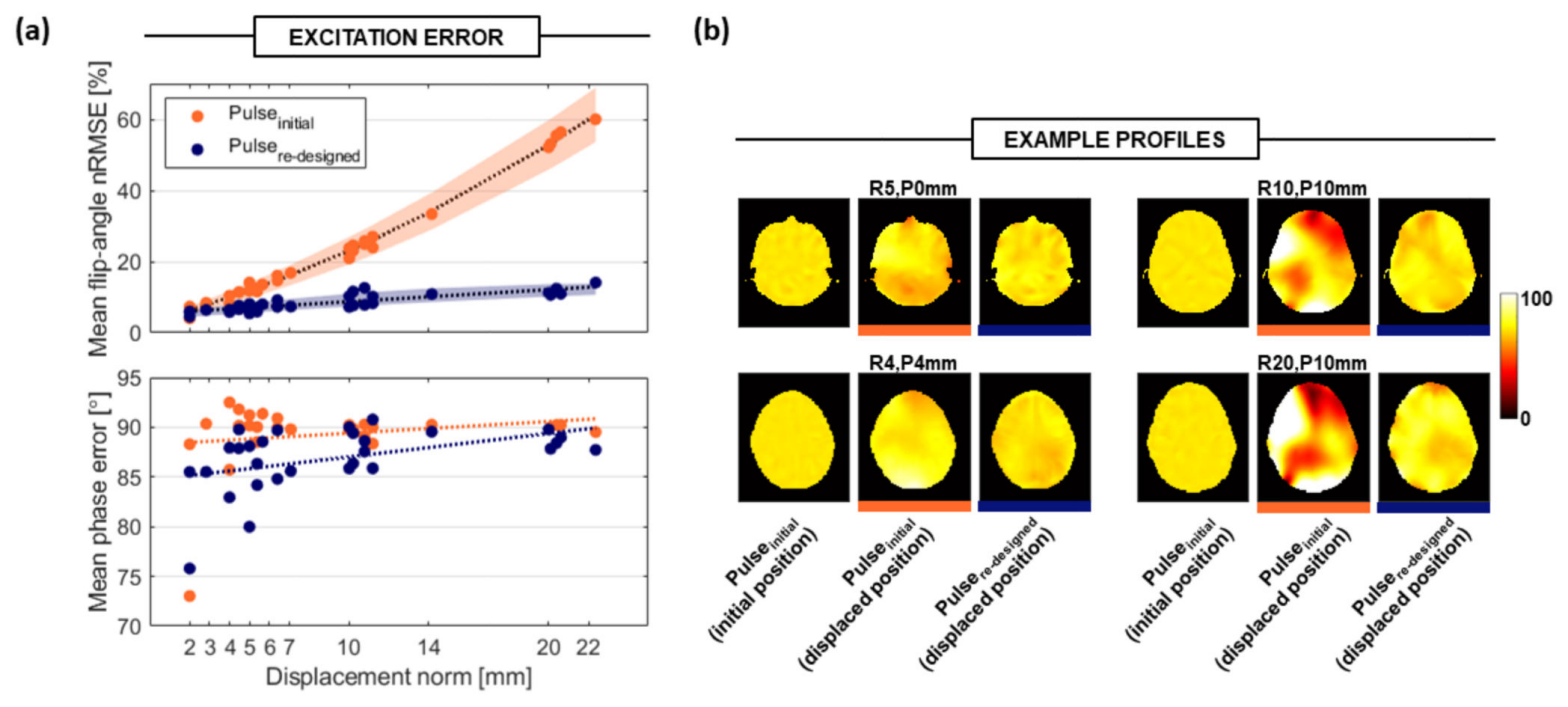
Excitation profile results for five-spoke pTx pulses following head motion. (A) Mean flip-angle nRMSE (above) and phase RMSE (below) for excitation profiles, averaged over slices and initial positions for each evaluated displacement. Excitation pulses were five-spoke pTx pulses designed using either the initial position (pulse_initial_) or predicted (pulseredesigned) B_1_ maps. The SD is shown as shaded regions for magnitude but is omitted for phase for clarity, as values were similar. (B) Example flip-angle profiles produced by pulse_initial_ at the initial position, by pulse_initial_ at the displaced position, and by pulseredesigned at the displaced position.

Figure 8 shows that flip-angle nRMSE for pulse_initial_ was strongly position-dependent, reaching a maximum of 76% following a displacement of R20, P5 mm. Conversely, pulses redesigned using B1_predicted_ (pulseredesigned) produced much improved flip-angle profiles when evaluated at the displaced position, yielding nRMSE of 14% for the same displacement. Maximum pulseredesigned nRMSE was 17% (at the R2, P10 mm position), whereas this error value was exceeded by pulse_initial_ (i.e., without any correction) after displacements of just ≥ 5 mm. The largest errors occurred in inferior slice locations for both pulses (slice information not shown). Maximum motion-related error in the excitation profile’s phase (110.4°) was reduced by 7.8° when using pulse_redesigned_.

Figure 8B shows flip-angle profiles for both pulses following several example displacements. Supporting Information Figure S2A also shows flip-angle nRMSE for nine example displacements. It should be noted that flip-angle uniformity for pulse_redesigned_ could be further improved by including phase relaxation in the design (as was done for pulse_initial_); however, this would permit excitation phase to vary throughout the scan, causing reconstruction inconsistencies.

### 3.3 Cascading

**Figure 9:**
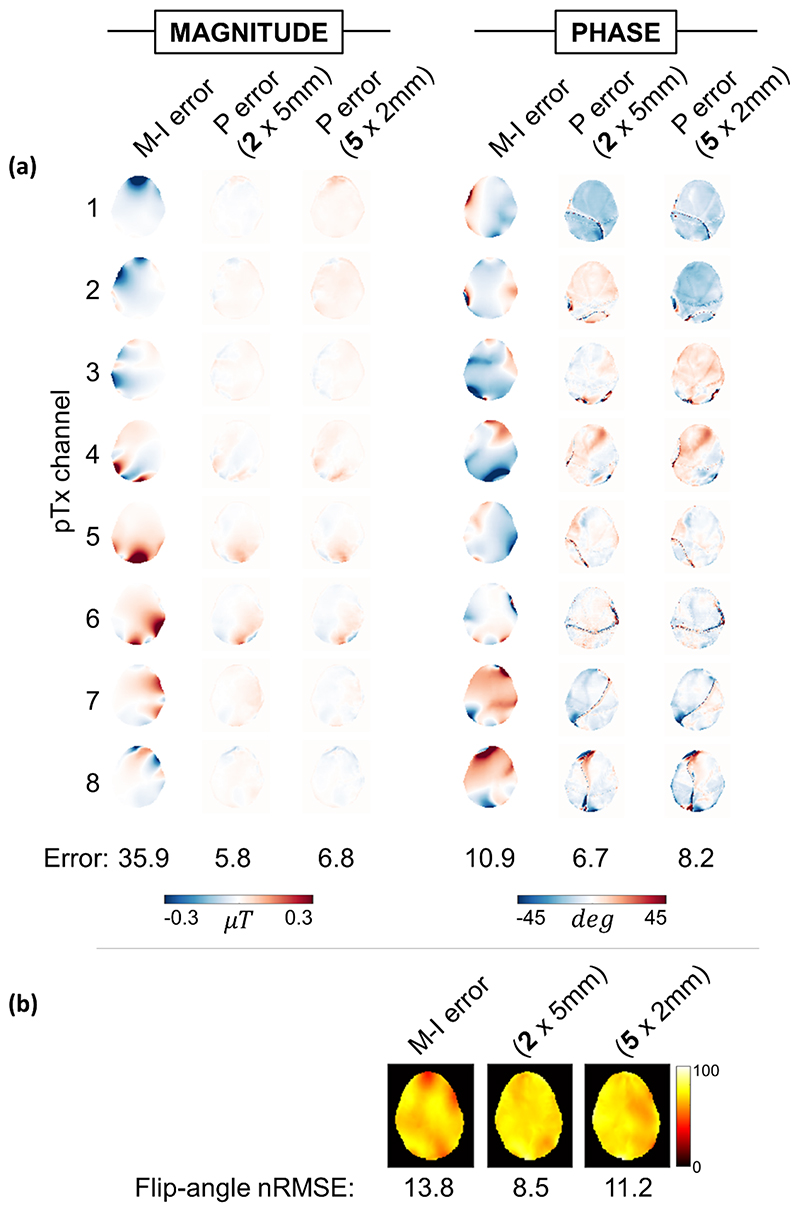
Effect of cascading the P2 mm network four times compared with cascading the P5 mm network once for evaluation at the R5, P10 mm position (along with the R5 mm network for the rightward component). (A) Example motion-induced (M-I) error and prediction (P) error for both cascade approaches for magnitude (left) and phase (right). Error shown below maps is nRMSE (%) for magnitude and L1 norm (°) for phase, both averaged over channels. (B) Comparison of flip-angle profiles and nRMSE for pulses designed using initial (left) and predicted maps using both cascade regimes (center and middle). Target flip angle is 70°.

The B1_predicted_ quality remained high when networks were cascaded multiple times; however, there was a weak linear relationship between prediction error and motion magnitude. To investigate the impact of cascading on prediction quality, we ran secondary analyses for displacements of R0, P10 mm, R-2, P10 mm, and R-5, P10 mm using only the P2 mm network for the posterior component. Running the 2 mm network five times (i.e., four cascades) led to approximate average increases in magnitude and phase error of 1.2% and 1.2°, respectively, compared with running the 5 mm network twice (one cascade). There was also reduced flip-angle uniformity compared with using the 5 mm network. Nevertheless, Figure 9 shows that motion-induced error was appreciably reduced using either approach.

### 3.4 Specific absorption rate

In addition to flip angle, SAR was also evaluated for the R-P grid positions. Following motion, psSAR produced by pulseredesigned was lower than that of pulse_initial_ in 89% of cases. For pulse_initial_, motion caused psSAR to increase (relative to that without motion; psSAR_center_) in 72% of evaluations. When pulse_redesigned_ was used instead, psSAR increased relative to the centered case in only 16% of the cases. The psSAR for nine example displacements is shown in Supporting Information Figure S2B.

**Figure 10:**
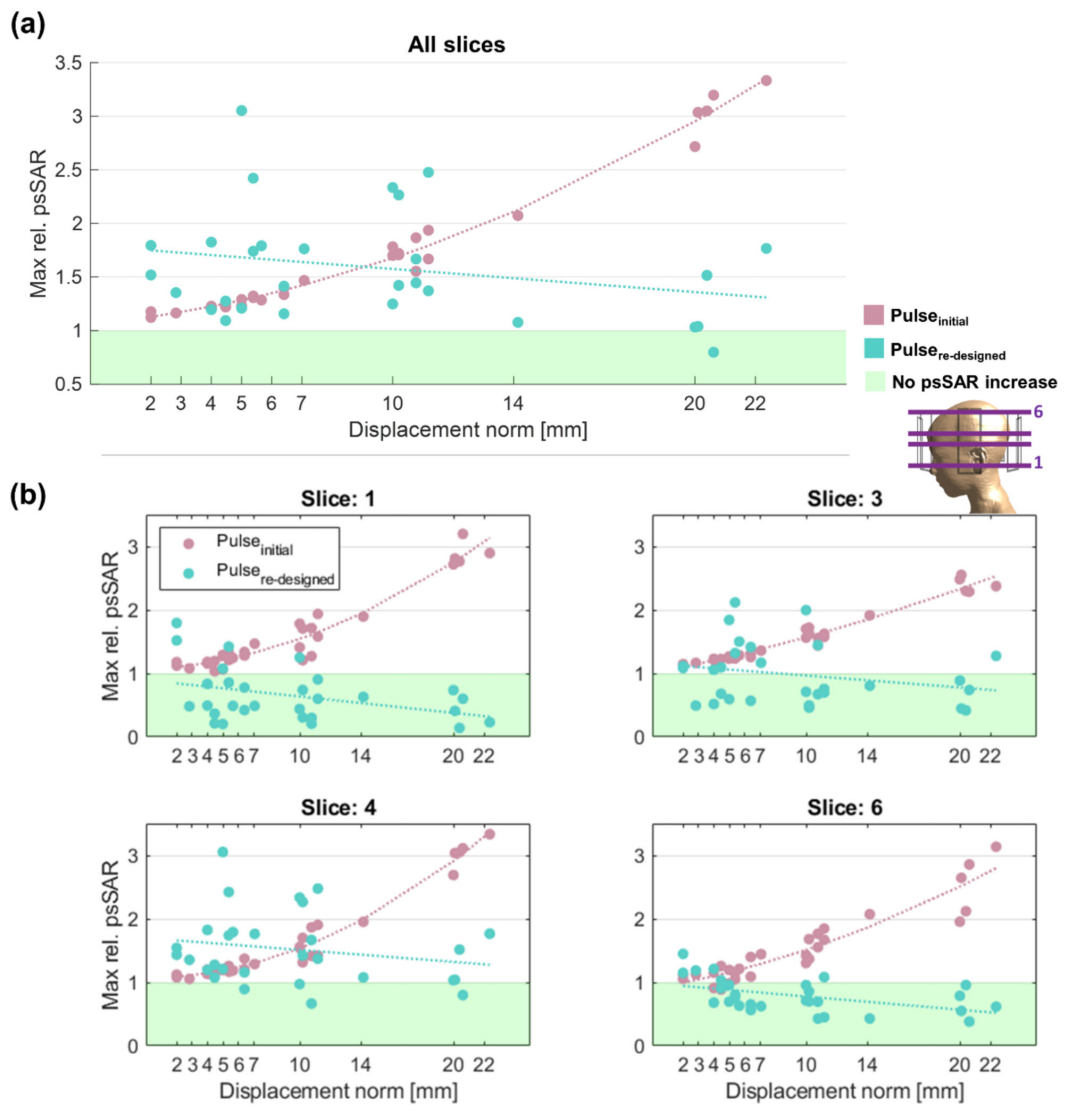
Peak 10g-averaged local SAR (psSAR) for pulse_initial_ and pulse_redesigned_ following motion (A) Worst-case psSAR for both pulses across all observed slice locations. (B) Slice-wise worstcase psSAR for both pulses. Slice locations are indicated in the inset on the right. Vertical axes show relative psSAR, calculated as psSAR as a factor of that without motion using pulse_initial_ (psSARcenter). The green-shaded region shows psSAR at or below psSAR_center_ (i.e., indicating that psSAR did not increase following motion). Neither pulse included SAR constraint in the design.

Figure 10A shows worst-case psSAR for each evaluated position (relative to psSARcenter). Figure 10B shows the same information, separated by slice location. Motion-related SAR change was similar across slices, whereas benefits offered by pulse_redesigned_ were most consistently seen in slices 1 and 6 (furthest from mid-axial locations). Pulse_redesigned_ yielded lower psSAR than pulse_initial_ following large displacements, but sometimes resulted in higher psSAR for small displacements, especially in slice 4 (mid-axial). In the worst observed case, pulse_redesigned_ yielded a 3.1-fold increase in psSAR (slice 4 at the posterior 5-mm position), whereas pulse_initial_ increased by a maximum of 3.3-fold (following the largest axial displacement). It should be noted that SAR was not used as a constraint in either pulse’s design.

## 4 Discussion

As far as we know, this study is the first to demonstrate motion-resolved B_1_ map prediction in pTx. We successfully trained a system of deep neural networks to estimate B_1_^+^ sensitivity distributions following simulated in-plane head motion. Predicted B_1_ maps were of sufficiently high quality to be used for tailored pTx pulse design, and resulting pulses outperformed conventionally designed tailored pulses under conditions of head motion.

Across the four R-P grid magnitude networks, prediction error was 4.5% ± 1.5% (mean ± SD) of the ground-truth B_1_ magnitude (tested individually without cascading and error calculated according to the definition in Abbasi-Rad et al^37^). This is in line with expectations based on previous work, in which magnitude B_1_ maps were predicted with mean error of 9.5 ± 7.1%.^37^ The experimental and 3D nature of data in Abbasi-Rad et al^37^ may have caused the slightly higher error. We also observe similar B1_predicted_ correlation coefficients to those reported in Wu et al^36^ (~0.99), which was retained even when our networks were cascaded multiple times. Yaw rotation caused higher error than translational motion, and prediction error was generally higher than that of translations. We attribute this to the much smaller training database used to train yaw networks.

It was previously reported that excitation profile nRMSE increases by approximately 2.4% per millimeter of axial displacement in simulations,^12^ and our results (2.7% per millimeter) are in good agreement. A 12%–22% flip-angle error was observed in vivo following motion in the range of about 5–16 mm in Kopanoglu et al., ^12^ and we observed an error of about 11%–35% for a similar range of motion. Error was reduced to approximately 8%–10% using the proposed approach in our study. For larger movements, the benefit of pulse redesign using predicted maps was even greater.

There was some slice dependence for both B1_predicted_ quality and flip-angle error, with the highest errors observed for inferior slice locations. This is in line with previous research, in which higher motion sensitivity was observed for inferior slices passing through the temporal lobes and the cerebellum.^12^ Inferior slices yield lower field magnitude, and B1_prediction_ quality was lower in regions with very low field magnitude, which may explain the residual slice dependence in predicted maps. An alternative explanation is that there were fewer inferior slices in the training data set compared with mid-axial slices, which could have resulted in better training for mid-axial slices.

Considering that the R5 mm training data included positions up to just 10 mm along the rightward axis, it is noteworthy that the R5 mm network was able to extrapolate beyond this by successfully cascading four times to estimate the fields at the R20 mm position. We attribute this to the global normalization, conducted over all data sets. Results for large displacements could likely be further improved by including extreme positions in the training data set.

Magnitude networks consistently outperformed those of phase. Prediction error exceeded motion-induced error for phase in about a third of evaluations. Improvements to excitation phase were evident but modest. It has been previously acknowledged that phase changes due to motion are difficult to model, and other work on motion-related field changes,^22^ including B_1_ prediction,^37^ often neglect phase altogether. Most of the phase-prediction error occurred at phase-wrapping boundaries. This was somewhat reduced by applying random phase offsets to pairs of input B_1_ maps, but it was not eliminated. In terms of pulse performance, these small, local errors are likely to have less impact than the global changes caused by motion, which could potentially accumulate when channels are superposed. Incidentally, when we instead trained networks using unwrapped phase data, the error in B1_predicted_ was globally higher and yielded inferior results compared with wrapped data. In contrast, the local prediction errors seen with wrapped data are not structured. We believe that the increase in error seen for unwrapped data was due to the increased dynamic range of unwrapped data, meaning that relative changes due to motion were smaller following normalization.

One limitation of this study is that it deals with simulated data only because of the lack of models for the RF coils at the institution due to proprietary information. In DeepQSM,^51^ training data were solely synthetic; the ground truth consisted of overlapping cubes and spheres with known (simulated) susceptibility values. This was convolved with a forward dipole kernel to create the corresponding input. Networks were able to resolve high-quality susceptibility maps for human brains, despite only being trained on simple geometric shapes. Similarly, Meladio et al. demonstrated successful in vivo validation following training with synthetic data.^52^ We believe that the method proposed here would be similarly generalizable if a realistic RF coil model (i.e., a model of the coil to be used) is used for simulations. Moreover, using simulated training data avoids the requirement for choreographed in vivo head movement to be replicated precisely across several subjects to create the training data sets, which would be practically infeasible. Aside from the initial (measured) input B_1_ map, networks automatically output maps in patient coordinates, making online registration unnecessary. However, motion tracking is needed to determine which network(s) are required, and for online corrections to gradient waveforms to update the imaging volume as in Zaitsev et al.^53^

The minimum motion resolution we consider here is 2 mm. Smaller movements could remain problematic for quantitative MR protocols that rely on signal changes on the scale of 1%–4% of the total signal.^21^ Finer discretization is possible through simulations of smaller displacements. The most appropriate motion discretization will depend on the user’s primary aims. While using finer discretization and cascading more (i.e., 5 × 2 mm vs 2 × 5 mm) did result in slightly higher error here, motion-induced error was still largely ameliorated.

In this study we considered only positive displacements (e.g., rightward, but not leftward). Because the trained networks are direction-specific, the same networks cannot handle negative displacements. This is due to the nature of inverting a deep neural network (not possible, as reversing convolutional layers results in a highly underdetermined problem). However, the training of networks for positive displacements does not limit generalizability, and additional networks can similarly be trained for negative displacements. In fact, by training a network for positive and negative displacements for each of the 6 degrees of freedom of motion, all rigid-body motion could be covered.

We focused on in-plane motion (axial translations and yaw rotation), as motion-induced error was observed to be more spatially varying. Although some motion-induced B_1_ error was observed in peripheral slices following inferior translation (see Supporting Information Figure S3), the error was relatively lower, and importantly, more global (i.e., spatially smoothly varying) within the slice. We attribute this to the fact that relative tissue-channel distances remain constant for through-slice translation. This means that simpler correction methods (e.g., slice-dependent pulse scaling) could feasibly be used to counteract the B_1_ effects of through-plane motion.

In contrast to B_1_, through-plane motion was shown to be more disruptive to B_0_ than within-plane motion.^54, 55^ Although B_0_ off-resonance can be incorporated in tailored pulse design,^56^ this cannot currently be updated in real time with pTx, as it increases the degrees of freedom to be optimized in the pulse, pushing redesign times beyond practically feasible TR values.^32^ Instead, motion-related effects on B_0_ can be corrected retrospectively, such as using data-driven coefficients to link motion with field changes.^55^ Alternatively, real-time B_0_ shimming may be possible with multicoil shim arrays by predicting B_0_ field changes due to motion in a manner similar to the method proposed here.

The SAR observations reported here are incidental. The focal point of this study was to develop a method to accurately estimate B_1_ maps following motion. Using SAR as a pulse design constraint would trade flip-angle homogeneity for reduced SAR, thereby overshadowing B_1_ quality. Hence, SAR was not used as a design constraint. Although motion-related SAR increase was generally lower for pulses redesigned with predicted B_1_ maps, it was higher for a minority of cases. Motion sensitivity of SAR in pTx has previously been reported to be similar across axial slices,^29^ and we also did not observe clear slice dependence for SAR motion sensitivity. However, we did observe that inferior-most and superior-most slices benefited from the proposed approach more consistently than mid-axial slices. Nevertheless, the overall improvement offered by pulseredesigned, especially for larger displacements, is promising for future development of this approach. Neural networks have previously been used to predict B_1_ maps for the purpose of SAR reduction.^37^ This was achieved through slice-wise pulse scaling based on a predicted 3D B_1_ magnitude. The entire 9-cm axial slab could be predicted within approximately 0.8 seconds, permitting concatenation into pseudo-3D B_1_ maps that could feasibly be used for pulse scaling or similar SAR management here. Pulse scaling based on B_1_^+^ cannot guarantee SAR compliance, as B_1_^+^ does not necessarily reflect electric-field distributions. However, SAR compliance could be ensured if (3D) electric fields were also predicted.

## 5 Conclusions

We have demonstrated a framework for a deep-learning approach for motion-resolved B_1_^+^ estimation in pTx. Estimated maps can be used for real-time tailored pulse redesign, yielding homogeneous flip-angle profiles in cases of head motion. Importantly, networks can be run sequentially to predict B_1_ maps following arbitrary displacements comprising multiple directions. Here, error was reduced for 35 displacements using networks trained for just five displacements. Our findings represent one potential avenue toward user-friendly, optimized pTx at 7 T.

## Supplementary Material

Supporting Information

## References

[1] Katscher U, Börnert P (2006). Parallel RF transmission in MRI. NMR Biomed.

[2] Deniz CM (2019). Parallel transmission for ultrahigh field MRI. Top Magn Reson Imaging.

[3] Grissom W, Yip C-Y, Zhang Z, Stenger VA, Fessler JA, Noll DC (2006). Spatial domain method for the design of RF pulses in multicoil parallel excitation. Magn Reson Med.

[4] Katscher U, Röhrs J, Börnert P (2005). Basic considerations on the im-pact of the coil array on the performance of Transmit SENSE. Magn Reson Mater Phys, Biol Med.

[5] Deniz CM, Vaidya MV, Sodickson DK, Lattanzi R (2016). Radiofrequency energy deposition and radiofrequency power requirements in parallel transmission with increasing distance from the coil to the sample. Magn Reson Med.

[6] De Greef M, Ipek O, Raaijmakers AJE, Crezee J, Van Den Berg CAT (2013). Specific absorption rate intersubject variability in 7T parallel transmit MRI of the head. Magn Reson Med.

[7] Gras V, Vignaud A, Amadon A, Le Bihan D, Boulant N (2017). Universal pulses: a new concept for calibration- free parallel transmission. Magn Reson Med.

[8] Gras V, Mauconduit F, Vignaud A (2018). Design ofuniversal parallel- transmit refocusing kT-point pulses and application to 3D T2-weighted imaging at 7T. Magn Reson Med.

[9] Tomi-Tricot R, Gras V, Thirion B (2019). SmartPulse, a machine learning approach for calibration- free dynamic RF shimming: preliminary study in a clinical environment. Magn Reson Med.

[10] Herrler J, Liebig P, Gumbrecht R (2021). Fast online-customized (FOCUS) parallel transmission pulses: a combination of uni-versal pulses and individual optimization. Magn Reson Med.

[11] Kopanoglu E (2021). Patient specific parallel transmit pulses are patient position dependent while safety models are fixed: safety implications.

[12] Kopanoglu E, Plumley A, Erturk MA, Deniz C, Wise R (2019). Implications of within-scan patient head motion on B1+ homogeneity and specific absorption rate at 7T.

[13] Bammer R, Zhang B, Deng W, Wiggins G, Stenger AV, Sodickson D (2010). Impact of motion on parallel transmission.

[14] Schön N, Petzold J, Seifert F (2020). Impact of respiration on B1+ field and SAR distribution at 7T using a novel EM simulation setup.

[15] Zaitsev M, Maclaren JH, Herbst M (2015). Motion artifacts in MRI: a complex problem with many partial solutions. J Magn Reson Imaging.

[16] Greene DJ, Church JA, Dosenbach NU (2016). Multivariate pattern classification of pediatric Tourette syndrome using functional connectivity MRI. Developmental Science.

[17] Schwarz ST, Afzal M, Morgan PS, Bajaj N, Gowland PA, Auer DP (2014). The ‘swallow tail’ appearance of the healthy nigrosome— a new accurate test of Parkinson’s disease: a case-control and retrospective cross-sectional MRI study at 3T. PLoS One.

[18] Saccà V, Sarica A, Quattrone A, Rocca F, Quattrone AN, Novellino F (2021). Aging effect on head motion: a machine learning study on resting state fMRI data. J Neurosci Methods.

[19] Chen KT, Salcedo S, Chonde DB (2018). MR- assisted PET motion correction in simultaneous PET/MRI studies of dementia sub-jects. J Magn Reson Imaging.

[20] Kecskemeti S, Samsonov A, Velikina J (2018). Robust motion correction strategy for structural MRI in unsedated children demonstrated with three-dimensional radial MPnRAGE. Radiology.

[21] Faraji-Dana Z, Tam F, Chen JJ, Graham SJ (2016). A robust method for suppressing motion- induced coil sensitivity variations during prospective correction of head motion in fMRI. Magn Reson Imaging.

[22] Wallace TE, Afacan O, Waszak M, Kober T, Warfield SK (2019). Head motion measurement and correction using FID navigators. Magn Reson Med.

[23] Vaughan J, Garwood M, Collins C (2001). 7T vs. 4T: RF power, homogeneity, and signal- to-noise comparison in head images. Magn Reson Med.

[24] Bazin P-L, Nijsse HE, Van Der Zwaag W (2020). Sharpness in motion corrected quantitative imaging at 7T. NeuroImage.

[25] Afacan O, Wallace TE, Warfield SK (2020). Retrospective correction of head motion using measurements from an electromagnetic tracker. Magn Reson Med.

[26] An H, Shin H-G, Ji S (2021). DeepResp: deep learning solution for respiration- induced B0 fluctuation artifacts in multi-slice GRE. NeuroImage.

[27] Gallichan D, Marques JP, Gruetter R (2016). Retrospective correction of involuntary microscopic head movement using highly ac-celerated fat image navigators (3D FatNavs) at 7T. Magn Reson Med.

[28] Deniz CM, Alon L, Brown R, Sodickson DK, Zhu Y (2012). Specific absorption rate benefits of including measured electric field in-teractions in parallel excitation pulse design. Magn Reson Med.

[29] Kopanoglu E, Deniz CM, Erturk MA, Wise RG (2020). Specific absorption rate implications of within-scan patient head motion for ultra- high field MRI. Magn Reson Med.

[30] Ajanovic A, Hajnal J, Malik S (2020). Positional sensitivity of specific absorption rate in head at 7T.

[31] Meliadò EF, Sbrizzi A, Van Den Berg CAT, Steensma BR, Luijten PR, Raaijmakers AJE (2020). Conditional safety margins for less conservative peak local SAR assessment: a probabilistic approach. Magn Reson Med.

[32] Kopanoglu E (2018). Near real- time parallel- transmit pulse design.

[33] Vinding MS, Aigner CS, Schmitter S, Lund TE (2021). DeepControl:2DRF pulses facilitating inhomogeneity and B0 off- resonance compensation in vivo at 7T. Magn Reson Med.

[34] DiGiacomo P, Maclaren J, Aksoy M (2020). A within-coil optical prospective motion-correction system for brain imaging at 7T. Magn Reson Med.

[35] Bortolotti L, Mougin OB (2020). R. Measurement of head motion using a field camera in a 7T scanner.

[36] Wu Y, Ma Y, Du J, Xing L (2020). Accelerating quantitative MR imaging with the incorporation of B1 compensation using deep learning. Magn Reson Imaging.

[37] Abbasi-Rad S, O’Brien K, Kelly S (2021). Improving FLAIR SAR efficiency at 7T by adaptive tailoring of adiabatic pulse power through deep learning estimation. Magn Reson Med.

[38] Goodfellow IJ, Pouget-Abadie J, Mirza M (2014). Generative adversarial nets.

[39] Gosselin M-C, Neufeld E, Moser H (2014). Development of a new generation of high- resolution anatomical models for medical device evaluation: the Virtual Population 3.0. Phys Med Biol.

[40] Wolf S, Diehl D, Gebhardt M, Mallow J, Speck O (2013). SAR simulations for high- field MRI: how much detail, effort, and accuracy is needed?. Magn Reson Med.

[41] Graesslin I, Homann H, Biederer S (2012). A specific absorption rate prediction concept for parallel transmission MR. Magn Reson Med.

[42] Standard III (2017). Determining the peak spatial- average specific absorption rate (SAR) in the human body from wireless com-munications devices, 30 MHz to 6 GHz— Part 1: General requirements for using the finite-difference time-domain (FDTD) method for SAR calculations. IEC/IEEE.

[43] Abadi M, Agarwal A, Barham P (2015). TensorFlow: Large-Scale Machine Learning on Heterogeneous Systems.

[44] Isola P, Zhu J-Y, Zhou T, Efros AA (2017). Image-to- image translation with conditional adversarial networks. arXiv.

[45] Ronneberger O, Fischer P, Brox T (2015). U-net: convolutional networks for biomedical image segmentation. Medical Image Computing and Computer-Assisted Intervention – MICCAI 2015 arXiv.

[46] Radford A, Metz L, Chintala S (2016). Unsupervised Representation Learning with Deep Convolutional Generative Adversarial Networks. arXiv.

[47] Kingma D, Lei BJ (2015). Adam: a method for stochastic optimization. arXiv.

[48] Grissom WA, Setsompop K, Hurley SA, Tsao J, Velikina JV, Samsonov AA (2017). Advancing RF pulse design using an open- competition format: report from the 2015 ISMRM challenge. Magn Reson Med.

[49] Kopanoglu E, Constable RT (2015). Radiofrequency pulse design using nonlinear gradient magnetic fields. Magn Reson Med.

[50] Setsompop K, Wald L, Alagappan V, Gagoski B, Adalsteinsson E (2008). Magnitude least squares optimization for parallel radio fre-quency excitation design demonstrated at 7 Tesla with eight channels. Magn Reson Med.

[51] Bollmann S, Rasmussen KGB, Kristensen M (2019). DeepQSM— using deep learning to solve the dipole inversion for quantita-tive susceptibility mapping. NeuroImage.

[52] Meliadò E, Raaijmakers A, Sbrizzi A (2020). A deep learning method for image- based subject-specific local SAR assessment. Magn Reson Med.

[53] Zaitsev M, Dold C, Sakas G, Hennig J, Speck O (2006). Magnetic resonance imaging of freely moving objects: prospective real- time motion correction using an external optical motion tracking system. NeuroImage.

[54] Liu J, de Zwart JA, van Gelderen P, Murphy-Boesch J, Duyn JH (2018). Effect of head motion on MRI B0 field distribution. Magn Reson Med.

[55] Brackenier Y, Lucilio C-G, Tomi-Tricot R (2021). Data-driven motion-corrected brain MRI incorporating pose dependent B0 fields.

[56] Wu X, Adriany G, Ugurbil K, Van de Moortele P-F (2013). Correcting for strong eddy current induced B0 modulation enables two- spoke RF pulse design with parallel transmission: demonstration at 9.4T in the human brain. PLoS One.

