# Supplementary material for: Rigid motion-resolved B1+ prediction using deep learning for real-time parallel-transmission pulse design
Source: Magn Reson Med. Author manuscript; Available in PMC 2022 Jul 17. (PMC7613077; doi:10.1002/mrm.29132)
Supplement: Supporting Information [file EMS149424-supplement-Supporting_Information.pdf]

7 SUPPORTING INFORMATION

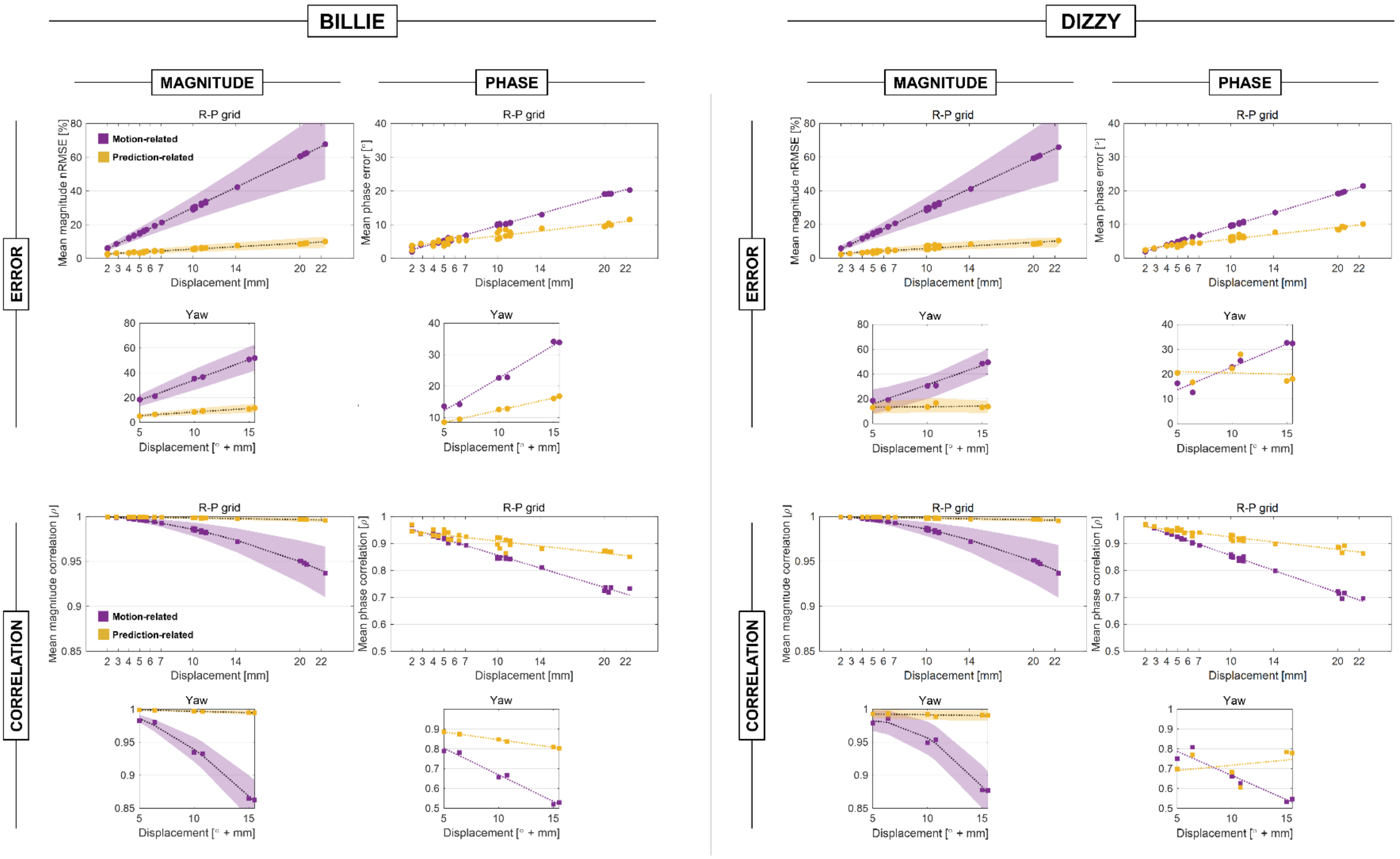

***Supporting Figure S1**: **(a)** B1-prediction quality (error and correlation) for the Billie model only. **(b)** B1-prediction quality (error and correlation) for the Dizzy model only. (Figure 5 displays the same evaluations but averaged over the two body models.) Data from the Billie model was used for validation during the training process, while the Dizzy model was not involved at all during training.*

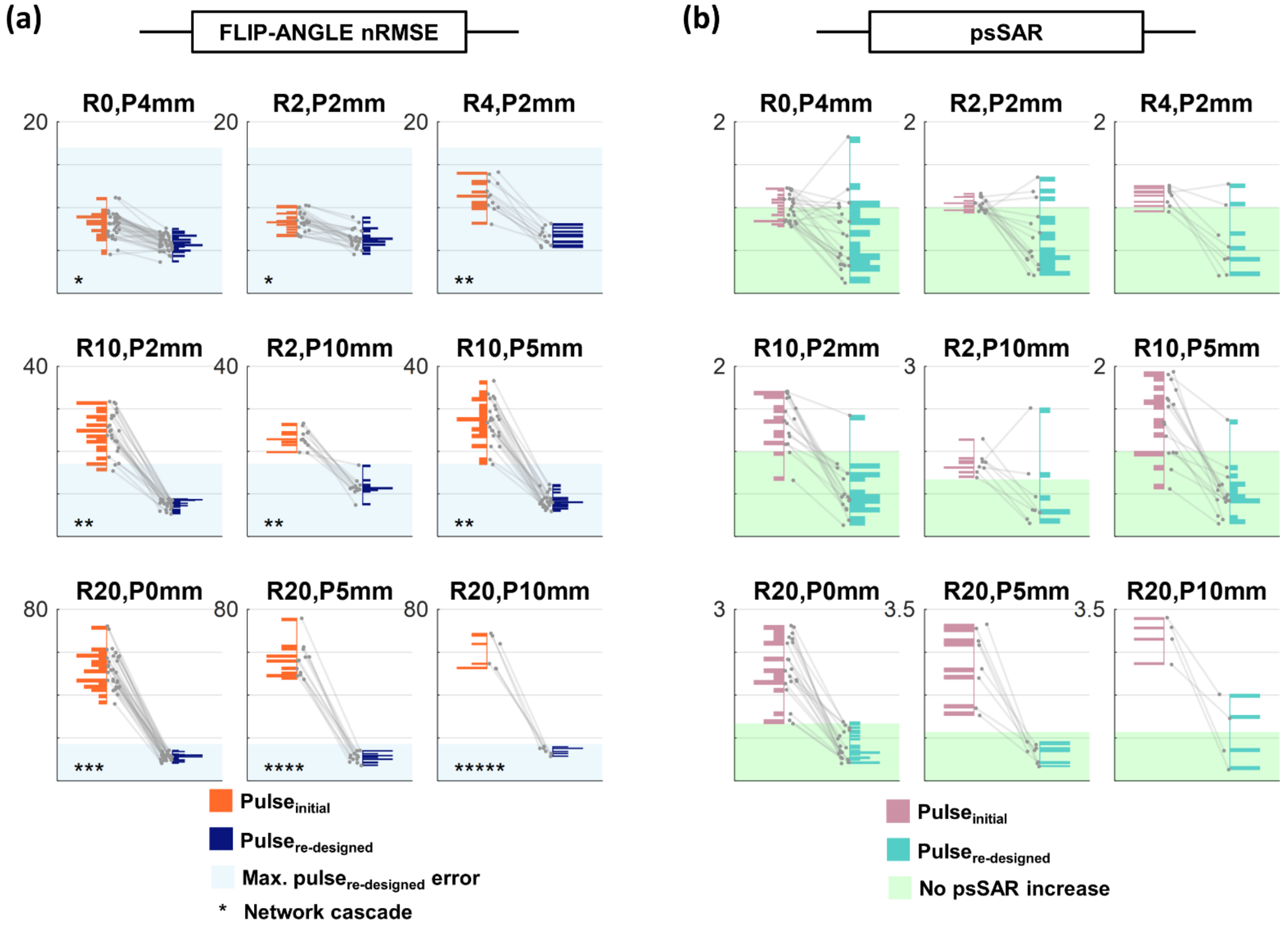

Supporting Information Figure S2**:** ***(a)*** *Raincloud plots showing flip-angle nRMSE (% of target flip-angle) for 9 example displacements using pulse$_{inital}$ (orange) and pulse$_{re\text{-}designed}$ (dark blue). Asterisks indicate the number of network cascades required for evaluation. The light blue shaded region shows maximum prediction nRMSE across all 35 evaluated displacements (consistent across panels).* ***(b)*** *All SAR evaluations for the same 9 example displacements. Vertical axes show relative psSAR, calculated as psSAR as a factor of that without motion using pulse$_{initial}$ (psSARcentre). The green shaded region shows psSAR at or below psSAR$_{centre}$ (i.e., indicating that psSAR did not increase following motion). Neither pulse included SAR-constraint in the design.*

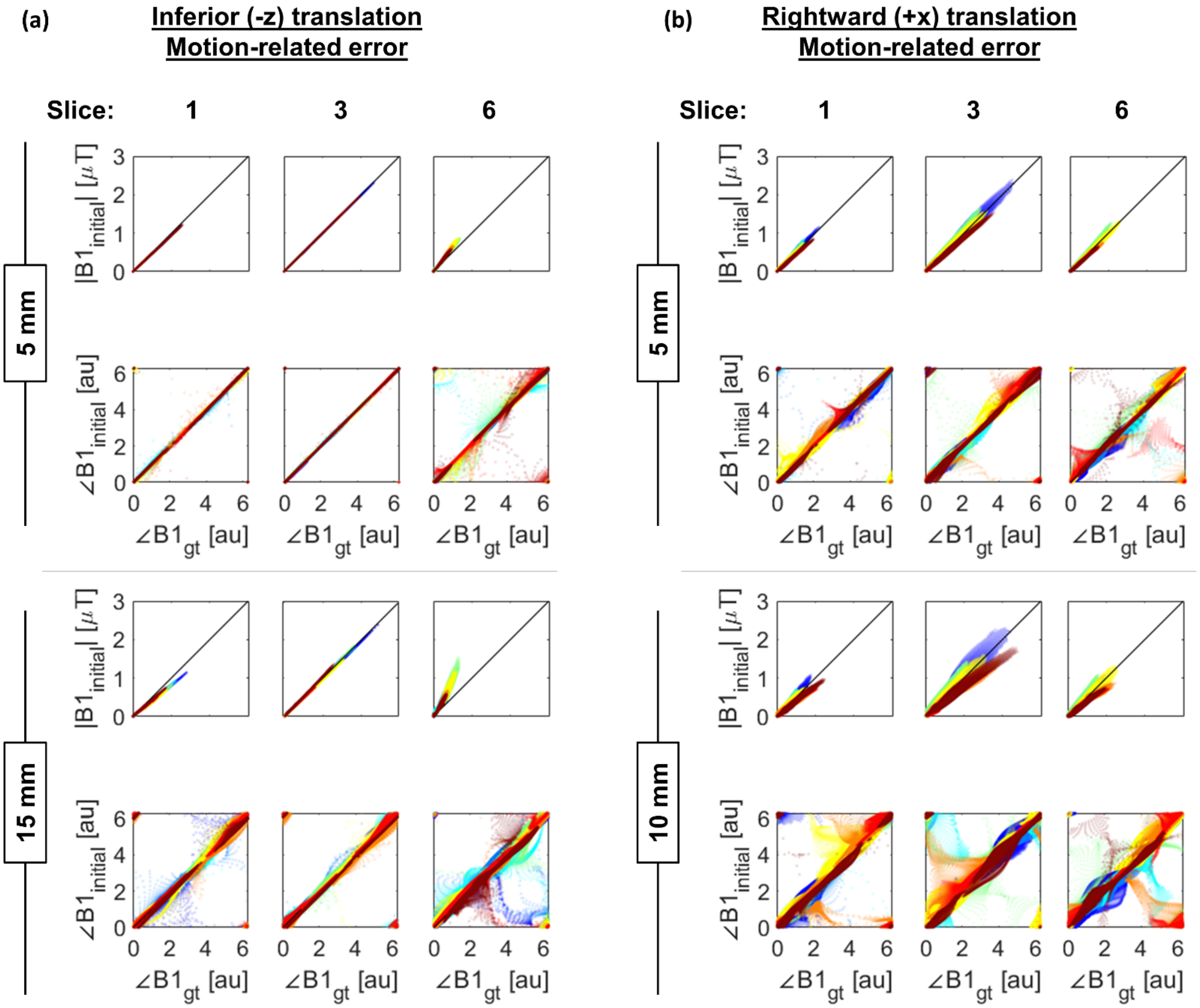

Supporting Information Figure S3: *Voxelwise correlations between initial ($B1_{initial}$) and ground-truth ($B1_{gt}$) B1 maps, to compare motion-related error following through-plane* ***(a)*** *and within-plane* ***(b)*** *displacements. Results are shown for two positions, and three slice locations (1 = least superior, 6 = most superior). Note that the large inferior displacement (15 mm) is greater than the large rightward displacement (10 mm), and yet error remains lower due to the through-plane nature of the movement.*
